# CENP-C and CENP-I are key connecting factors for kinetochore and CENP-A assembly

**DOI:** 10.1242/jcs.180786

**Published:** 2015-12-15

**Authors:** Nobuaki Shono, Jun-ichirou Ohzeki, Koichiro Otake, Nuno M. C. Martins, Takahiro Nagase, Hiroshi Kimura, Vladimir Larionov, William C. Earnshaw, Hiroshi Masumoto

**Affiliations:** 1 Laboratory of Cell Engineering, Department of Frontier Research, Kazusa DNA Research Institute, 2-6-7 Kazusa-Kamatari, Kisarazu, Chiba 292-0818, Japan; 2 Division of Biological Science, Graduate School of Science, Nagoya University, Furo-cho, Chikusa-ku, Nagoya, Aichi 464-8602, Japan; 3 Wellcome Trust Centre for Cell Biology University of Edinburgh, King's Buildings, Mayfield Road, Edinburgh EH9 3JR, UK; 4 Public Relations Team, Kazusa DNA Research Institute, 2-6-7 Kazusa-Kamatari, Kisarazu, Chiba 292-0818, Japan; 5 Department of Biological Sciences, Graduate School of Bioscience and Biotechnology, Tokyo Institute of Technology, Yokohama 226-8501, Japan; 6 Developmental Therapeutic Branch, National Cancer Institute, National Institutes of Health, Bethesda, MD, USA

**Keywords:** CENP-A, CENP-C, CENP-I, Centromere, Human artificial chromosome, HAC, Kinetochore

## Abstract

Although it is generally accepted that chromatin containing the histone H3 variant CENP-A is an epigenetic mark maintaining centromere identity, the pathways leading to the formation and maintenance of centromere chromatin remain unclear. We previously generated human artificial chromosomes (HACs) whose centromeres contain a synthetic alpha-satellite (alphoid) DNA array containing the tetracycline operator (alphoid^tetO^). We also obtained cell lines bearing the alphoid^tetO^ array at ectopic integration sites on chromosomal arms. Here, we have examined the regulation of CENP-A assembly at centromeres as well as *de novo* assembly on the ectopic arrays by tethering tetracycline repressor (tetR) fusions of substantial centromeric factors and chromatin modifiers. This analysis revealed four classes of factors that influence CENP-A assembly. Interestingly, many kinetochore structural components induced *de novo* CENP-A assembly at the ectopic site. We showed that these components work by recruiting CENP-C and subsequently recruiting M18BP1. Furthermore, we found that CENP-I can also recruit M18BP1 and, as a consequence, enhances M18BP1 assembly on centromeres in the downstream of CENP-C. Thus, we suggest that CENP-C and CENP-I are key factors connecting kinetochore to CENP-A assembly.

## Abstract (precis)

**Highlighted Article:** Tethering analysis using various centromere and kinetochore factors, and chromatin modifiers reveals that CENP-C and CENP-I are key connecting factors for kinetochore and CENP-A assembly.

## INTRODUCTION

The kinetochore directs accurate chromosome segregation by controlling chromosome movements through interactions with spindle microtubules, and also by serving as a platform for various regulatory pathways. Kinetochores assemble on centromere chromatin marked by nucleosomes containing the centromere-specific histone H3 variant CENP-A (Allshire and Karpen, 2008; Earnshaw and Rothfield, 1985). The interphase centromere complex (ICEN) associates with the CENP-A nucleosome (Izuta et al., 2006; Obuse et al., 2004), and the constitutive-centromere-associated network (CCAN) forms the inner kinetochore (Basilico et al., 2014; Cheeseman and Desai, 2008; Foltz et al., 2006; Gascoigne et al., 2011; Hori et al., 2008; Okada et al., 2006). The CCAN factors CENP-C (Saitoh et al., 1992) and CENP-T act as a crucial platform for the kinetochore during mitosis (Gascoigne et al., 2011; Hori et al., 2008, 2013; Nishino et al., 2013; Przewloka et al., 2011; Rago et al., 2015). CENP-C binds to CENP-A nucleosomes (Carroll et al., 2010; Kato et al., 2013), thereby acting as a hub for kinetochore formation. CENP-C recruits the Mis12 complex, which then recruits the NDC80 complex (Cheeseman et al., 2006; Petrovic et al., 2010; Przewloka et al., 2011; Screpanti et al., 2011) and CENP-T also recruits the NDC80 complex (Schleiffer et al., 2012). The KMN network, composed of KNL1, the Mis12 complex and the NDC80 complex is the main microtubule-binding component of the outer kinetochore (Cheeseman et al., 2006; DeLuca et al., 2006).

The CENP-A nucleosome assembly exhibits a number of surprises. The number of CENP-A nucleosomes is reduced by half through DNA replication and not replenished until mitosis is completed (Jansen et al., 2007). Why centromeres go through mitosis with half the maximal number of CENP-A nucleosomes is not known. Several factors including the Mis18 complex (Mis18α–Mis18β–M18BP1) and the CENP-A-specific histone chaperone HJURP are involved in this replenishment of CENP-A nucleosomes (Barnhart et al., 2011; Dunleavy et al., 2009; Foltz et al., 2009; Fujita et al., 2007; Hayashi et al., 2004; Shuaib et al., 2010). Among these factors, M18BP1 has been reported to interact with CENP-C (Dambacher et al., 2012; Moree et al., 2011). In addition to these factors, which are involved in CENP-A replenishment during early G1 phase, the remodeling and spacing factor (RSF) (Perpelescu et al., 2009) and small GTPase MgcRacGAP (also known as RACGAP1) act during mid to late G1 phase to stabilize newly assembled CENP-A (Lagana et al., 2010).

Recent studies have demonstrated that chromatin states also affect CENP-A assembly. We have previously introduced synthetic alphoid DNAs containing the tetO motif in every second monomer (alphoid^tetO^) into culture cells and established a synthetic human artificial chromosome (the alphoid^tetO^-HAC) carrying a functional centromere (Nakano et al., 2008). Subsequent studies have used this HAC to probe the chromatin state that is permissive for CENP-A assembly and maintenance. Induction of histone H3K9me3 heterochromatin by tethering a fusion protein comprising the Tet repressor protein (tetR) (which binds to the alphoid^tetO^ sequence) and the KRAB-AB silencing domain of Kid-1 (also known as ZNF354A), KAP1 (the repressive scaffold recruited by the TetR and Kid-1 silencing domain fusion; also known as TRIM28) or the H3K9 histone methyltransferase (HMT) Suv39h1 to the alphoid^tetO^-HAC all reduce CENP-A assembly dramatically on the alphoid^tetO^-HAC (Cardinale et al., 2009; Nakano et al., 2008; Ohzeki et al., 2012). Removal of the transcription-associated mark H3K4me2 by tethering the demethylase LSD1 also reduced CENP-A assembly (Bergmann et al., 2011). In contrast, tethering the catalytic domain of histone acetyltransferase (HAT) p300 (also known as EP300) or PCAF induced assembly of expressed HA-tagged CENP-A on the alphoid^tetO^-HAC and at a ectopic alphoid^tetO^ integration site in a chromosomal arm (Ohzeki et al., 2012). To date, many factors (e.g. CCAN and ICEN) have been shown to be localized in centromeric chromatin by biochemical fractionation and mass spectrometry as described above. For many of the factors identified in these pulldowns, it remains unclear whether they also contribute to CENP-A assembly or not.

Here, we have exploited a synthetic biology, or tethering, analysis to probe the ability of a number of kinetochore components and chromatin factors to recruit CENP-A to the HAC centromere and to assemble CENP-A *de novo* at an ectopic alphoid^tetO^ array on a chromosomal arm. From these analyses, we classified the factors into four groups that increase or decrease CENP-A assembly on the alphoid^tetO^ array. Surprisingly, tethering of outer kinetochore components of the KMN network can induce *de novo* CENP-A assembly on the ectopic array. This assembly proceeds through recruitment of CENP-C, which then recruits M18BP1 to promote *de novo* CENP-A assembly. Moreover, we found that CENP-I can also recruit M18BP1 and, as a consequence, enhances M18BP1 assembly at centromeres, in a process that acts downstream of CENP-C. CENP-C and CENP-I are, thus, revealed to be key factors connecting the outer kinetochore structure through the KMN network to promote epigenetic maintenance of CENP-A chromatin through M18BP1.

## RESULTS

### Identification of factors that increase or decrease CENP-A assembly on the HAC kinetochore

**Fig. 1.**
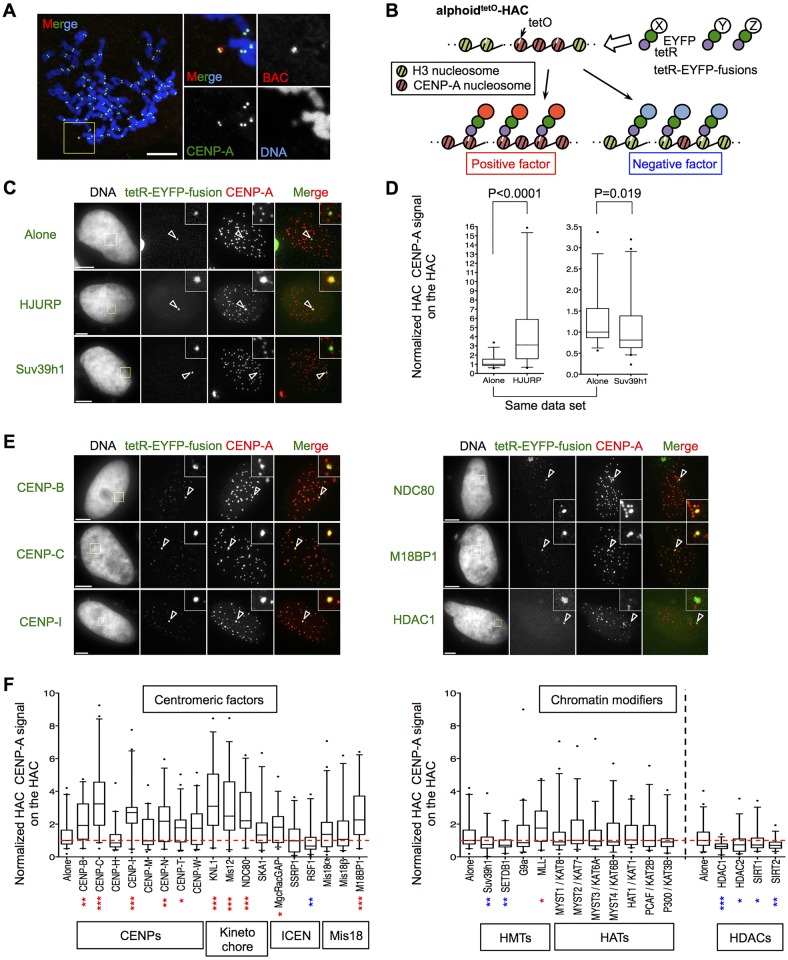
**Identification of factors that increase or decrease CENP-A assembly on the HAC kinetochore.** (A) Examples of the HeLa cell line containing a stable alphoid^tetO^-HAC (HeLa-HAC-2-4). Mitotic cells were spread on cover glass and stained with DAPI (blue), anti-CENP-A antibody (green) and the BAC DNA probe (red) (Ohzeki et al., 2012). Magnified images of the area enclosed by a yellow line are shown in the right panels. Scale bar: 10 μm. (B) A schematic drawing of the tetR-EYFP–alphoid^tetO^ array tethering system on alphoid^tetO^-HAC. X, Y and Z represent candidate proteins. (C,E) Representative images of HeLa-HAC-2-4 cells transfected with indicated tetR-EYFP fusion proteins (green). Cells were stained with DAPI and anti-CENP-A (red) at 48 h after transfection. Arrowheads indicate the alphoid^tetO^-HAC. Scale bars: 5 μm. (D,F) Normalized CENP-A signals on the HAC after tethering tetR-EYFP fusion proteins were quantified at 48 h after transfection. The HAC-associated CENP-A signal was normalized to the average of centromere signals on the endogenous centromeres in the same nucleus (Fig. S1). Relative values to the median of control (Alone) (red dashed line) are shown in a boxplot (*n*=23–49 cells). Alternative names are indicated by a solidus (‘/’). The box represents the 25–75th percentiles, and the median is indicated. The whiskers show the 5–95th percentiles. *P<0.05; **P<0.01; ***P<0.001 from control (Mann–Whitney test; red, increase; blue, decrease).

To evaluate factors that modulate CENP-A assembly, we have adopted a synthetic biology, or tethering, approach using the alphoid^tetO^-HAC, which segregates comparably to endogenous chromosomes (HeLa-HAC-2-4; Ohzeki et al., 2012) (Fig. 1A). By using tetR-EYFP, we tethered various fusion proteins to the alphoid^tetO^ array and subsequently quantified CENP-A levels on the HAC by indirect immunofluorescence (Fig. 1B; Fig.S1A). As controls, we tethered the CENP-A-specific chaperone HJURP, as a positive regulator, and the H3K9 methyltransferase Suv39h1, as a negative regulator (Fig. 1C). Tethering tetR-EYFP–HJURP significantly increased the CENP-A signal on the HAC, whereas tethering tetR-EYFP–Suv39h1 caused a corresponding decrease (Fig. 1D). These changes in CENP-A levels on the HAC were confirmed using chromatin immunoprecipitation quantitative PCR (ChIP-qPCR) analysis (Fig. S1B).

We next applied this approach by tethering a number of centromeric factors to the alphoid^tetO^-HAC as tetR-EYFP fusions (Fig. 1E,F). Tethering of CENP-C, CENP-I, CENP-N, CENP-T and KMN network components, all of which are structural components of the kinetochore, increased CENP-A levels on the HAC. So did tethering of MgcRacGAP and CENP-B, both of which have been reported to be involved in stabilizing CENP-A nucleosomes (Fachinetti et al., 2015; Fujita et al., 2015; Lagana et al., 2010). Thus, many centromeric factors were found to regulate the CENP-A assembly positively on the HAC. The Mis18 complex is involved in priming centromeres for CENP-A assembly. Interestingly, tethering of M18BP1 increased CENP-A levels on the alphoid^tetO^-HAC, but tethering of Mis18α and Mis18β did not.

When we applied this approach using a number of chromatin modifiers (Fig. 1F), tethering of transcriptional silencers, such as HMTs Suv39h1 and SETDB1, significantly decreased CENP-A signals on the HAC, consistent with previous reports (Cardinale et al., 2009; Nakano et al., 2008; Ohzeki et al., 2012). Similarly, tethering a range of histone deacetylases (HDACs), including HDAC1, HDAC2, SIRT1 and SIRT2 (Hassig and Schreiber, 1997) also decreased CENP-A signals on the alphoid^tetO^-HAC. In contrast, tethering the H3K4 HMT MLL (also known as KMT2A) (Dou et al., 2005) increased CENP-A levels on the HAC. Previous studies have shown that H3K4me2 is required for CENP-A assembly on the alphoid^tetO^-HAC (Bergmann et al., 2011). Interestingly, tethering of the HATs MYST1, MYST2, MYST3, MYST4, HAT1, PCAF (also known as KAT8, KAT7, KAT6A, KAT6B, KAT1 and KAT2B, respectively) and p300, did not significantly change the CENP-A levels on the HAC centromere.

### Identification of the factors that can induce *de novo* CENP-A assembly

**Fig. 2.**
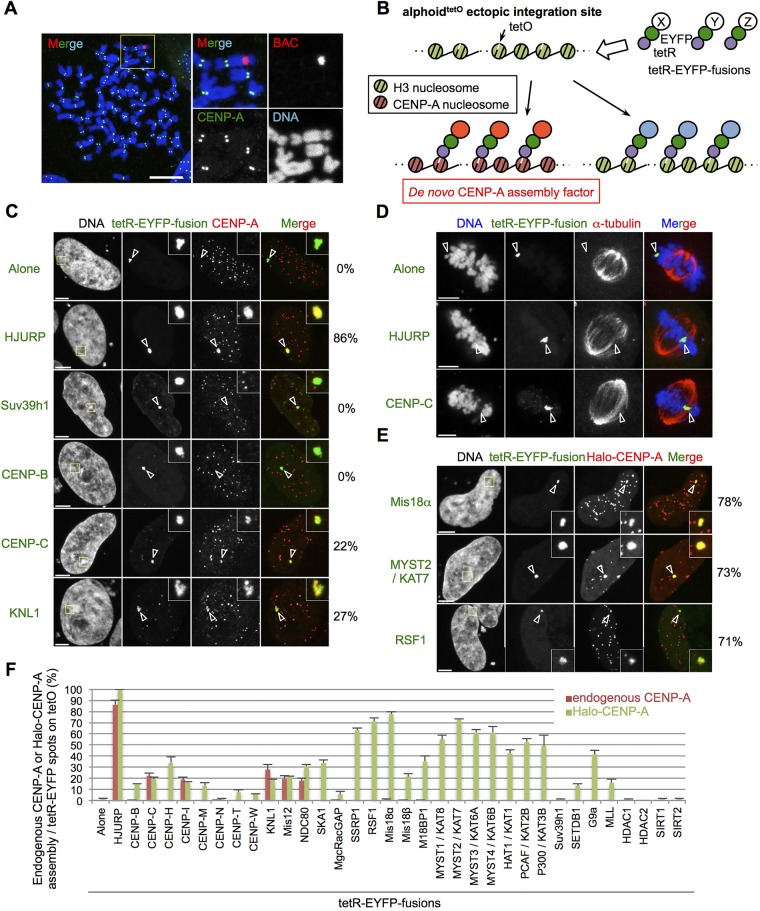
**Identification of the factors that can induce *de novo* CENP-A assembly.** (A) Examples of the HeLa cell line containing the ectopic alphoid^tetO^ integration site (HeLa-Int-03). Mitotic cells were spread on cover glass and stained with DAPI (blue), anti-CENP-A antibody (green) and BAC DNA probe (red). Magnified images of the area enclosed by a yellow line are shown in right panels. Scale bar: 10 μm. (B) A schematic drawing of the tetR-EYFP–alphoid^tetO^ array tethering system on the ectopic site. X, Y and Z represent candidate proteins. (C) Representative images of HeLa-Int-03 cells transfected with the indicated tetR-EYFP fusion proteins (green). Cells were stained with DAPI and anti-CENP-A (red) at 48 h after transfection. The frequency of *de novo* endogenous CENP-A assembly on the ectopic site (arrowheads) is shown on the right of the images. Scale bars: 5 μm. (D) Representative images of HeLa-Int-03 cells transfected with tetR-EYFP alone, tetR-EYFP–CENP-C or tetR-EYFP–HJURP (green). Cells were stained with DAPI and anti-α-tubulin (red) at 48 h after transfection. Arrowheads indicate the ectopic site. Scale bars: 5 μm. (E) Representative images of HeLa-Int-03 cells co-transfected with the indicated tetR-EYFP fusion proteins (green) and Halo–CENP-A. Cells were stained with DAPI and the Halo-tag TMR ligand (red) 24 h after transfection. The frequency of *de novo* Halo–CENP-A assembly on the ectopic site (arrowheads) is shown on the right of the images. Scale bars: 5 μm. (F) Frequency of *de novo* assembly of endogenous CENP-A or Halo–CENP-A on the ectopic site at 48 or 24 h after transfection, respectively. Endogenous CENP-A or Halo–CENP-A signals on tetR-EYFP spots as a percentage of the total tetR-EYFP spots in each sample (*n*=100 cells for endogenous CENP-A, *n*=50 cells for Halo–CENP-A). Alternative names are indicated by a solidus (‘/’). Results are mean±s.e.m. (*n*=3 experiments).

Among the factors that regulate CENP-A assembly positively at the alphoid^tetO^-HAC centromere, HJURP has been previously reported to induce *de novo* CENP-A assembly when tethered to non-centromeric sites on chromosomal arms (Barnhart et al., 2011; Bassett et al., 2012; Ohzeki et al., 2012). We therefore tested whether the tethering of tetR-EYFP fusion proteins to a non-centromeric alphoid^tetO^ integration site on a chromosomal arm covered with heterochromatin (HeLa-Int-03; Ohzeki et al., 2012) could induce *de novo* CENP-A assembly (Fig. 2A,B).

In controls, tethering of tetR-EYFP or tetR-EYFP–Suv39h1 did not induce *de novo* CENP-A assembly. As expected, tethering of tetR-EYFP–HJURP induced *de novo* CENP-A assembly in 86% of cells (Fig. 2C). Interestingly, tethering of CENP-C, CENP-I and KMN network components induced robust *de novo* CENP-A assembly on the ectopic site (Fig. 2C,F). These results on inducing CENP-A assembly by the tethering of CENP-C and CENP-I using human cells (HeLa) are consistent with the previous findings using chicken DT40 cells (Hori et al., 2013). The HJURP or the CENP-C tethering resulted in capturing bundles with an excess amount of microtubules at the ectopic site in the mitotic phase, suggesting the assembly of kinetochore components (Fig. 2D). In contrast, tethering of CENP-B, CENP-T, CENP-N, MgcRacGAP and MLL did not induce *de novo* CENP-A assembly at the heterochromatic ectopic site even though they all increased CENP-A assembly at the centromere of the alphoid^tetO^-HAC (Fig. 1F, Fig. 2C,F). Although the Mis18 complex is normally involved in CENP-A assembly, tethering of M18BP1, Mis18α and Mis18β did not significantly induce *de novo* CENP-A assembly on the ectopic site.

All of the above studies looked at assembly of endogenous CENP-A on alphoid^tetO^ arrays. Remarkably different results were obtained when we tethered the various components to the array in cells that were also overexpressing Halo-tagged CENP-A (Halo–CENP-A). Expression of Halo–CENP-A enhanced *de novo* CENP-A assembly at the ectopic site to 100% of cells following the tethering of tetR-EYFP–HJURP. Halo–CENP-A overexpression was not sufficient to induce CENP-A assembly following the tethering of tetR-EYFP alone or tetR-EYFP–Suv39h1 (1–2% of cells) (Ohzeki et al., 2012) (Fig. 2F). However, in cells overexpressing Halo–CENP-A a wide range of factors were found to induce substantial CENP-A assembly. These included Mis18α, Mis18β and M18BP1, the HATs MYST1, MYST2, MYST3, MYST4, HAT1, PCAF and p300 as well as CENP-H, RSF1 and SSRP1 (Fig. 2E,F).

**Fig. 3.**
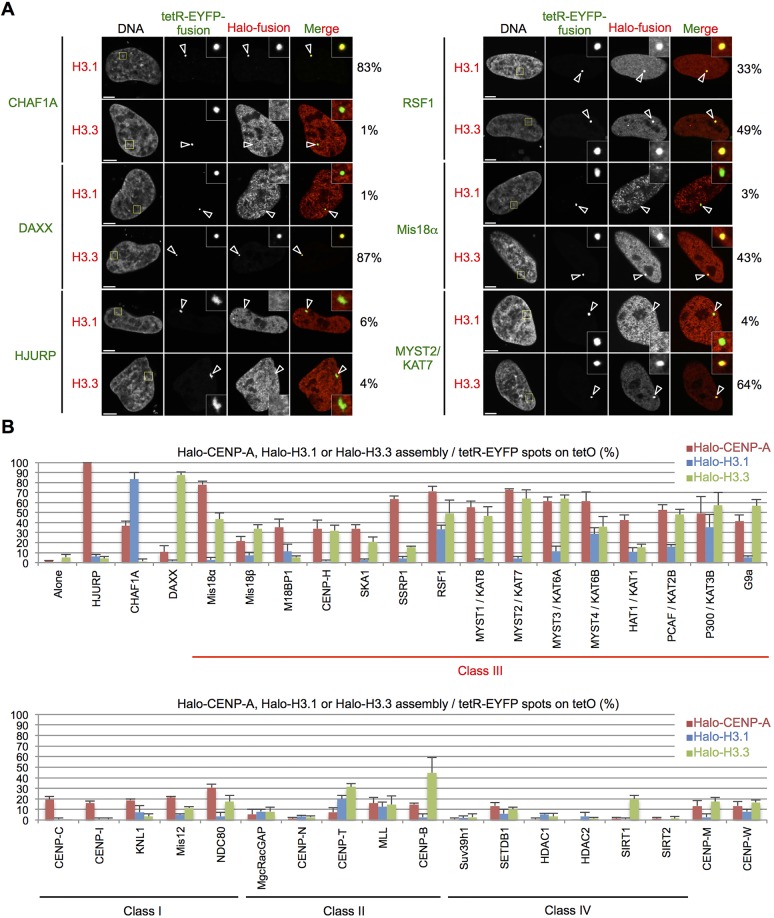
**Halo–H3.1 or Halo–H3.3 assembly on the ectopic site.** (A) Representative images of HeLa-Int-03 cells co-transfected with indicated tetR-EYFP fusion proteins (green) and Halo–H3.1 or Halo–H3.3. Cells were stained with DAPI and the Halo-tag TMR ligand (red) 24 h after transfection. The frequency of Halo–H3.1 or Halo–H3.3 assembly on the ectopic site (arrowheads) is shown on the right of the images. Scale bars: 5 μm. (B) Frequency of Halo–CENP-A (Fig. 2F), Halo–H3.1 or Halo–H3.3 assembly on the ectopic site 24 h after transfection. Our classification of each factor is shown in Fig. 4. Halo–CENP-A, Halo–H3.1 or Halo–H3.3 signals on tetR-EYFP spots as a percentage of the total tetR-EYFP spots in each sample (*n*=50 cells). Alternative names are indicated by a solidus (‘/’). Results are mean±s.e.m. (*n*=3 experiments).

As HATs, RSF1 and SSRP1 are general chromatin factors (LeRoy, 1998), so we thought that these factors might also induce the assembly of canonical histone H3. Thus, we performed similar experiments in cells overexpressing Halo–H3.1 or Halo–H3.3 instead of Halo–CENP-A (Fig. 3A,B). Tethering tetR-EYFP–CHAF1A or tetR-EYFP–DAXX, the specific histone chaperones for H3.1 (replication dependent) or H3.3 (replication independent), respectively (Drane et al., 2010; Kaufman et al., 1995), resulted in robust assembly of Halo–H3.1 or Halo–H3.3 on the alphoid^tetO^ array in 83% and 87% of cells, respectively. Importantly, this result indicates that the specificities of each chaperone for its respective H3 variant are retained even in this tethering assay.

Most factors that induced Halo–CENP-A assembly also induced high levels of Halo–H3.3 assembly on the ectopic site. Intriguingly, tetR-EYFP–Mis18α and –Mis18β also efficiently induced H3.3 assembly in 30–40% of cells. It is possible that common mechanisms might be involved in Halo–CENP-A assembly and Halo–H3.3 assembly when this class of factors is tethered to the alphoid^tetO^ array.

### Classification of tested factors based on the influences on CENP-A assembly

**Fig. 4.**
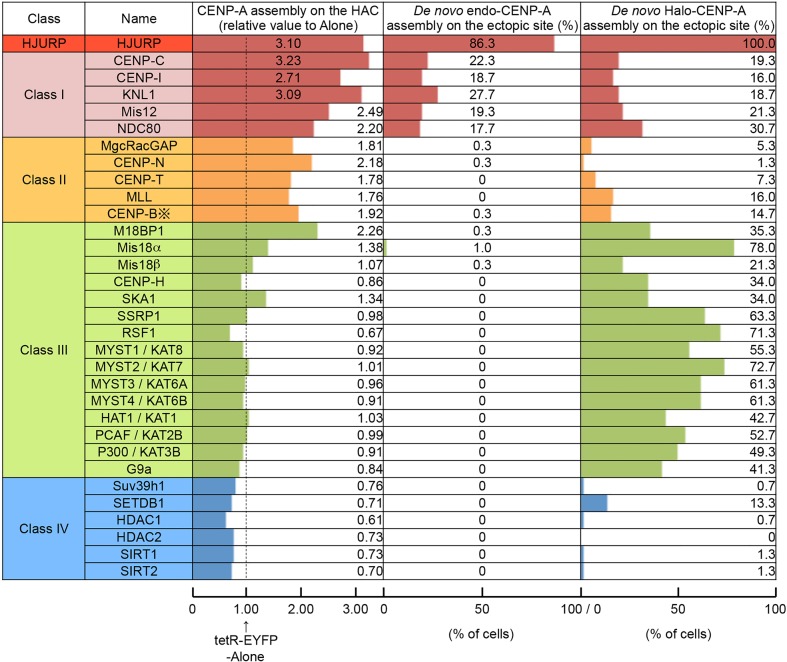
**Classification of tested factors.** Numbers on the left column show the median value of the CENP-A signal relative to the control (tetR-EYFP alone) on the HAC centromere (Fig. 1F). Numbers in the middle and right columns show the percentage of *de novo* endogenous CENP-A or Halo–CENP-A assembly at the ectopic site (Fig. 2F). Note that endogenous CENP-B also binds to CENP-B box, the recognition sequence for CENP-B, on the HAC and ectopic site (※) (Ohzeki et al., 2012). Alternative names are indicated by a solidus (‘/’).

These three analyses to CENP-A assembly allowed us to divide the tested factors into four classes (Fig. 4). The first group, class I factors, increase endogenous CENP-A assembly on the HAC and induce *de novo* CENP-A assembly at the ectopic site. These factors include CENP-C, CENP-I, KNL1, Mis12 and NDC80. We excluded HJURP from class I because it is known to directly bind to CENP-A as a chaperone. The second group, class II factors, increase endogenous CENP-A assembly on the HAC centromere, but do not induce efficient *de novo* CENP-A assembly when tethered to the ectopic site even when CENP-A is overexpressed. These factors include MgcRacGAP, CENP-N, CENP-T, MLL and CENP-B. Class III factors induce efficient *de novo* CENP-A assembly (≥20%) when tethered to the ectopic site provided that CENP-A is overexpressed. These factors include M18BP1, Mis18α, Mis18β, CENP-H, SKA1, RSF1, SSRP1, MYST1, MYST2, MYST3, MYST4, HAT1, PCAF, p300 and G9a. Finally, class IV factors decrease endogenous CENP-A assembly on the HAC and do not induce efficient *de novo* CENP-A assembly by their tethering even when CENP-A is overexpressed. These factors include Suv39h1, SETDB1, HDAC1, HDAC2, SIRT1 and SIRT2. Most of the factors involved in transcriptional silencing fall into class IV.

### CENP-C is the crucial factor for *de novo* CENP-A assembly

As our classification revealed, class I factors are sufficient for *de novo* CENP-A assembly when tethered to the ectopic site. Interestingly, all class I factors are involved in the kinetochore (Cheeseman et al., 2006; Nishihashi et al., 2002; Okada et al., 2006). This suggests that there might be a link between the kinetochore and *de novo* CENP-A assembly.

**Fig. 5.**
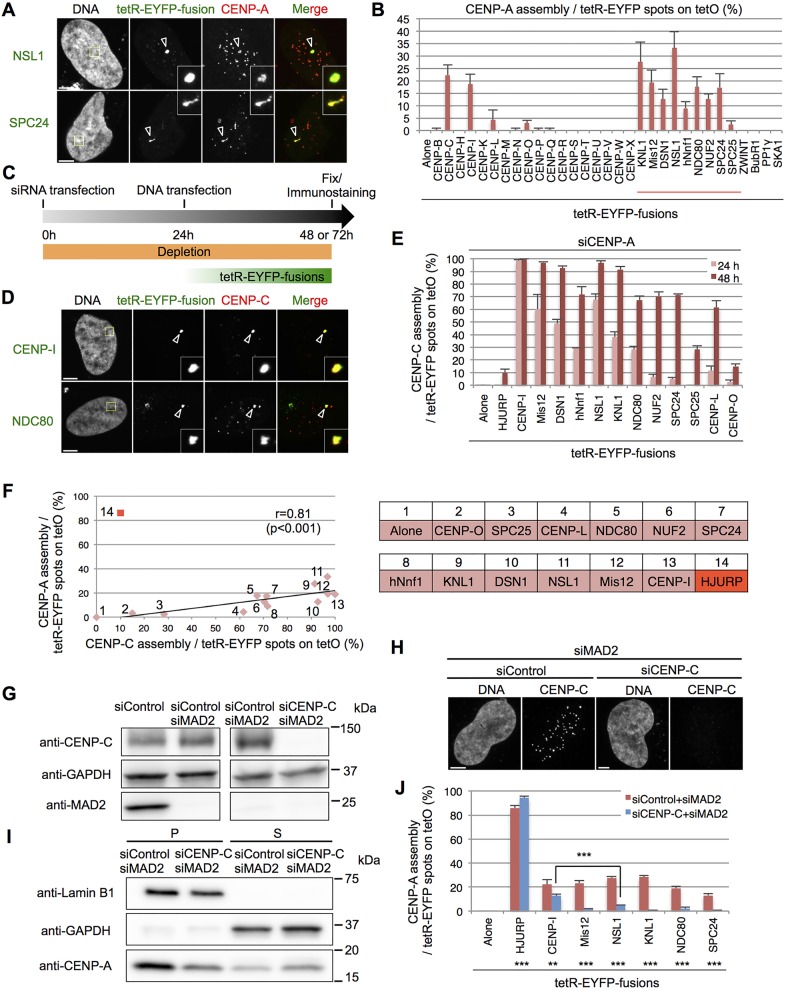
**CENP-C is required for *de novo* CENP-A assembly induced by the class I kinetochore components.** (A) Representative images of HeLa-Int-03 cells transfected with tetR-EYFP–NSL1 or tetR-EYFP–SPC24 (green). Cells were stained with DAPI and anti-CENP-A (red) at 48 h after transfection. Arrowheads indicate the ectopic site. Scale bars: 5 μm. (B) Frequency of *de novo* CENP-A assembly on the ectopic site at 48 h after transfection. CENP-A signals on tetR-EYFP spots as a percentage of the total tetR-EYFP spots in each sample (*n*=100 cells). The red line shows KMN network components. Data shown in Fig. 2F are included for comparison. Results are mean±s.e.m. (*n*=3 experiments). (C) Schematic for the experiments shown in D,E and J. HeLa-Int-03 cells were first transfected with siRNA. After 24 h incubation, tetR-EYFP fusion expression vectors were transfected. Cells were stained with DAPI and each antibody. (D) Representative images of HeLa-Int-03 cells transfected with the indicated tetR-EYFP fusion proteins (green) after transfection with siRNA against CENP-A (siCENP-A). Cells were stained with DAPI and anti-CENP-C antibody (red) at 48 h after plasmid transfection. Arrowheads indicate the ectopic site. Scale bar: 5 μm. (E) Frequency of CENP-C assembly on the ectopic site determined as described in C. CENP-C signals on tetR-EYFP spots as a percentage of the total tetR-EYFP spots in each sample (*n*=100 cells) fixed at 24 or 48 h after plasmid transfection. Results are mean±s.e.m. (*n*=3 experiments). (F) Correlation between *de novo* CENP-A (from B) and CENP-C (from E; 48h) assembly. tetR-EYFP fusion proteins that correspond to the numerals in the graph are shown on the right. The correlation coefficient (*r*) and *P* value were calculated (except for tetR-EYFP–HJURP) and are shown in the graph. (G) CENP-C and MAD2 expression were analyzed by immunoblotting using antibodies against CENP-C, GAPDH (loading control) and MAD2. Cells were harvested at 72 h after control (siControl), CENP-C (siCENP-C) or MAD2 (siMAD2) siRNA transfection. (H) Representative images of HeLa-Int-03 cells transfected with indicated siRNAs. Cells were stained with DAPI and anti-CENP-C at 72 h after transfection. Scale bar: 5 μm. (I) The amount of chromosomal (P) and soluble (S) CENP-A were analyzed by immunoblotting using antibodies against Lamin B1 (loading control for P), GAPDH (loading control for S) and CENP-A. Cells were harvested and fractionated at 72 h after siRNA transfection. (J) Frequency of *de novo* CENP-A assembly on the ectopic site under the indicated siRNAs transfection. CENP-A signals on tetR-EYFP spots as a percentage of the total tetR-EYFP spots in each sample (*n*=100 cells) fixed at 48 h after plasmid transfection. **P<0.01; ***P<0.001 for significant differences between indicated tetR-EYFP fusion proteins or siRNA sets (Fisher's exact test). Results are mean±s.e.m. (*n*=3 experiments).

To further explore this link, we explored the ability of several other kinetochore and CCAN components to induce *de novo* CENP-A assembly on the ectopic alphoid^tetO^ array. Interestingly, components of KMN network all efficiently induced *de novo* CENP-A assembly in this assay (Fig. 5A,B). In contrast to these structural components of the kinetochore, ZWINT, BubR1, PP1γ and SKA1 (Fig. 5B), which are thought to be involved in regulating kinetochore activity through the spindle assembly checkpoint, did not induce *de novo* CENP-A assembly.

**Fig. 6.**
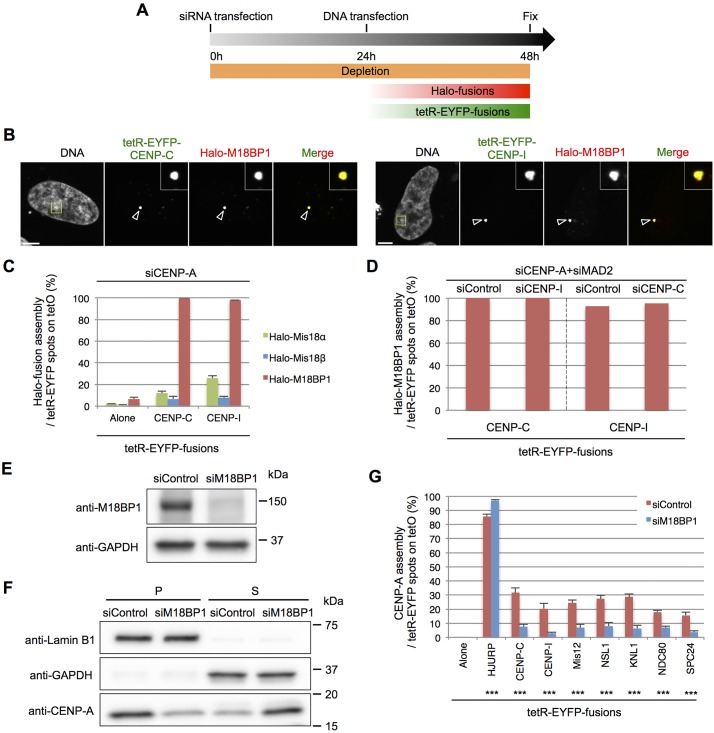
**CENP-C or CENP-I can independently recruit M18BP1.** (A) Schematic for the experiments shown in B, C and D. HeLa-Int-03 cells were first transfected with siRNA. After 24 h incubation, Halo fusion and tetR-EYFP fusion expression vectors were co-transfected. (B) Representative images of HeLa-Int-03 cells co-transfected with tetR-EYFP–CENP-C or tetR-EYFP–CENP-I (green) and Halo-M18BP1 after transfection with siRNA against CENP-A (siCENP-A). Cells were stained with DAPI and the Halo-tag TMR Ligand (red) at 24 h after plasmid transfection. Arrowheads indicate the ectopic site. Scale bars: 5 μm. (C) Frequency of each Halo fusion assembly on the ectopic site. Halo fusion signals on tetR-EYFP spots as a percentage of the total tetR-EYFP spots in each sample (*n*=50 cells) fixed at 24 h after plasmid transfection. Results are mean±s.e.m. (*n*=3 experiments). (D) Frequency of Halo–M18BP1 assembly on the ectopic site after transfection of control siRNA (siControl) and siRNA against CENP-A (siCENP-A), MAD2 (siMAD2), CENP-I (siCENP-I) and CENP-C (siCENP-C) as indicated. tetR-EYFP fusion proteins were co-transfected with Halo–M18BP1 after siRNA transfection. Halo-fusion signals on tetR-EYFP spots as a percentage of the total tetR-EYFP spots in each sample (*n*=50 cells) fixed at 24 h after plasmid transfection. Results are mean±s.e.m. (*n*=3 experiments). (E) M18BP1 expression was analyzed by immunoblotting using antibodies against M18BP1 and GAPDH (loading control). Cells were harvested at 72 h after each transfection with control and M18BP1 (siM18BP1) siRNA. (F) The amount of chromosomal (P) and soluble (S) CENP-A were analyzed by immunoblotting using antibodies against Lamin B1 (loading control for P), GAPDH (loading control for S) and CENP-A. Cells were harvested and fractionated at 72 h after each siRNA transfection. (G) Frequency of *de novo* CENP-A assembly on the ectopic site after transfection of the indicated siRNA. CENP-A signals on tetR-EYFP spots as a percentage of the total tetR-EYFP spots in each sample (*n*=100 cells) fixed at 48 h after plasmid transfection (72 h after siRNA transfection). Asterisks indicate significant differences from tetR-EYFP alone. ***P<0.001 (Fisher's exact test). Results are mean±s.e.m. (*n*=3 experiments).

**Fig. 7.**
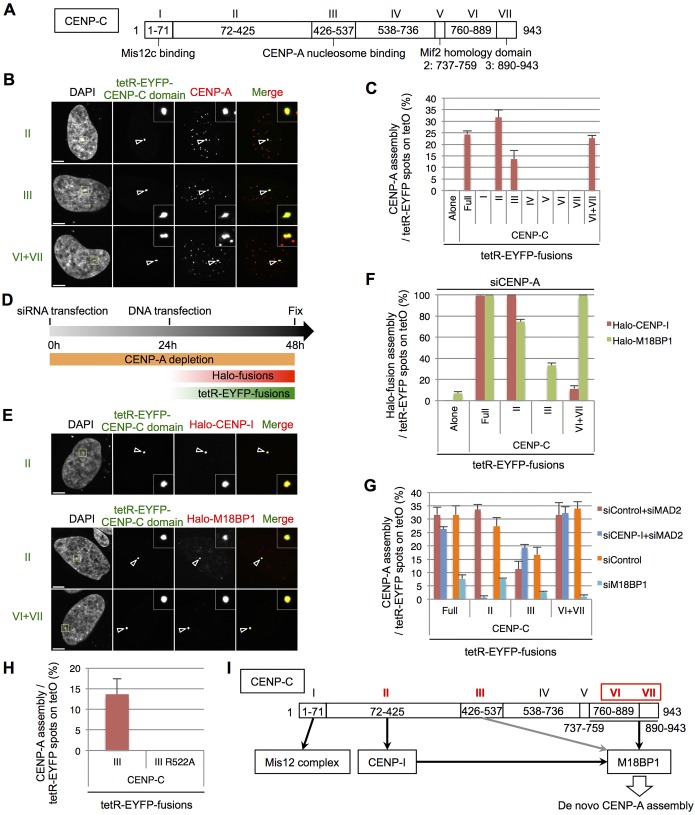
**CENP-C induces *de novo* CENP-A assembly through multiple pathways, including one through CENP-I.** (A) Schematics of CENP-C protein domains. (B) Representative images of HeLa-Int-03 cells transfected with the indicated tetR-EYFP–CENP-C domains. Cells were stained with DAPI and anti-CENP-A (red) at 48 h after transfection. Arrowheads indicate the ectopic site. Scale bars: 5 μm. (C) The frequency of *de novo* CENP-A assembly on the ectopic site. CENP-A signals on tetR-EYFP spots as a percentage of the total tetR-EYFP spots in each sample (*n*=100 cells) at 48 h after transfection. Results are mean±s.e.m. (*n*=3 experiments). (D) Schematic for the experiments shown in E and F. HeLa-Int-03 cells were first transfected with siRNA against CENP-A (siCENP-A). After 24 h incubation, Halo fusion and tetR-EYFP fusion expression vectors were co-transfected. (E) Representative images of HeLa-Int-03 cells co-transfected with the indicated tetR-EYFP fusion (green) and Halo-fusion proteins after siCENP-A transfection. Cells were stained with DAPI and the Halo-tag TMR Ligand (red) at 24 h after plasmid transfection. Arrowheads indicate the ectopic site. Scale bars: 5 μm. (F) Frequency of each Halo fusion assembly on the ectopic site. Halo fusion signals on tetR-EYFP spots as a percentage of the total tetR-EYFP spots in each sample (*n*=50 cells) at 24 h after plasmid transfection. Results are mean±s.e.m. (*n*=3 experiments). (G) Frequency of *de novo* CENP-A assembly on the ectopic site after transfection of control siRNA (siControl) or siRNA against MAD2 (siMAD2), CENP-I (siCENP-I) or M18MP1 (siM18BP1). CENP-A signals on tetR-EYFP spots as a percentage of the total tetR-EYFP spots in each sample (*n*=100 cells) at 48 h after plasmid transfection (72 h after siRNA transfection). Results are mean±s.e.m. (*n*=3 experiments). (H) HeLa-Int-03 cells were transfected with tetR-EYFP–CENP-C III WT and the R522A mutant, which prevents this domain binding to CENP-A nucleosomes (Carroll et al., 2010). The graph shows the frequency of *de novo* CENP-A assembly on the ectopic site. CENP-A signals on tetR-EYFP spots as a percentage of the total tetR-EYFP spots in each sample (*n*=100 cells). Results are mean±s.e.m. (*n*=3 experiments). (I) Summary of recruited factors and pathway of *de novo* CENP-A assembly from CENP-C. The red roman number denotes domains sufficient for *de novo* CENP-A assembly.

Using various deletion mutants of tetR-EYFP–NSL1 or tetR-EYFP–Mis12, both of which are Mis12 complex components and, hence, expected to interact with the KMN network and CENP-C (Petrovic et al., 2010; Screpanti et al., 2011), we found that NSL1 and Mis12 deletion mutants that had lost the ability to interact with CENP-C also lost the ability to induce *de novo* CENP-A assembly (Fig. S2A,B). We confirmed that NSL1 and Mis12 interact with CENP-C even following CENP-A depletion by small interfering RNA (siRNA)-mediated knockdown. This control minimizes the likelihood of possible secondary CENP-C recruitment through pre-assembled CENP-A (Fig. S2C–F; CENP-A depletions were also performed similarly in Figs 5, 6 and 7, and Fig. S3). We concluded that CENP-C recruited by tetR-EYFP–NSL1 or tetR-EYFP–Mis12 induces *de novo* CENP-A assembly on the ectopic site.

Remarkably, tethering of all class I factors to the ectopic alphoid^tetO^ array recruits CENP-C in the CENP-A depletion condition (Fig. 5C–E). These experiments revealed a strong correlation between the efficiency of CENP-C recruitment and *de novo* CENP-A assembly activity following the tethering of class I factors (Fig. 5F). These results suggest that CENP-C is a crucial factor for inducing *de novo* CENP-A assembly.

Interestingly, CENP-C recruited by tethering of tetR-EYFP fusion class I factors retains its binding to the ectopic site in the absence of CENP-A assembly even after dissociation of the tetR-EYFP fusion proteins by the addition of doxycycline (Fig. S3). In this case, *de novo* CENP-A assembly might be promoted by the remarkable increase of CENP-C recruitment that occurs after 24 h to 48 h (Fig. 5E).

### CENP-C and CENP-I can both induce *de novo* CENP-A assembly

To test whether CENP-C is essential for *de novo* CENP-A assembly, we tethered the main class I factors to the ectopic alphoid^tetO^ array in cells depleted of CENP-C (Fig. 5G–J). CENP-C depletion caused a mitotic arrest (Fukagawa and Brown, 1997), thus we performed a double depletion with MAD2 to release the mitotic arrest (Choi et al., 2011) (Fig. 5G; Fig. S4A,B). We confirmed that the level of soluble CENP-A did not decrease following CENP-C depletion (Fig. 5I). Under these conditions *de novo* CENP-A assembly induced by tethering its chaperone HJURP did not decrease (Fig. 5J). In contrast, induction of CENP-A assembly by tethering KMN network components dramatically decreased following CENP-C depletion. *De novo* CENP-A assembly induced by tethering CENP-I also decreased substantially, however, interestingly, considerable CENP-A assembly remained (Fig. 5J). This suggests that CENP-I can induce *de novo* CENP-A assembly without CENP-C. CENP-I and CENP-C do not act equivalently in inducing new CENP-A assembly. Indeed, CENP-I depletion had no effect on the *de novo* assembly of CENP-A induced by tethering KMN components to the ectopic alphoid^tetO^ array (Fig. S4C–G).

### CENP-C or CENP-I can independently recruit M18BP1

Taken together, the above results indicate that CENP-C and CENP-I independently recruit one or more factors that promote *de novo* CENP-A assembly. M18BP1 has been reported to interact with CENP-C (Dambacher et al., 2012; Moree et al., 2011), and the Mis18 complex is involved in the assembly of newly synthesized CENP-A at centromeres during early G1 phase (Fujita et al., 2007). We therefore asked whether tethered CENP-C and CENP-I recruit Halo–Mis18α, Halo–Mis18β or Halo–M18BP1 to the ectopic alphoid^tetO^ array (Fig. 6A–C). As expected, CENP-C tethering recruited Halo–M18BP1 in 100% of cells. Surprisingly, CENP-I tethering also recruited Halo–M18BP1 in 98% of cells. This recruitment of Halo–M18BP1 by CENP-C tethering did not decrease following siRNA depletion of CENP-I. Similarly, the recruitment of Halo–M18BP1 by CENP-I tethering also did not decrease following siRNA depletion of CENP-C (Fig. 6D). We conclude that CENP-C or CENP-I can independently recruit M18BP1.

In order to test whether M18BP1 is essential for CENP-A recruitment, we next tested *de novo* CENP-A assembly induced by tethering of tetR-EYFP fusion class I factors following M18BP1 depletion by siRNA (Fig. 6E–G). Importantly, this did not decrease the levels of soluble CENP-A or *de novo* CENP-A assembly induced by tethering HJURP. In contrast, we observed a dramatic decrease in *de novo* CENP-A assembly following the tethering of class I factors. Thus CENP-C and CENP-I induce *de novo* CENP-A assembly through M18BP1 recruitment.

### CENP-C induces *de novo* CENP-A assembly through multiple pathways, including one through CENP-I

CENP-C strongly interacts with CENP-I (Fig. 5D,E). It is therefore likely that CENP-C induces *de novo* CENP-A assembly through CENP-I recruitment. For further investigation of the role of CENP-C and CENP-I in *de novo* CENP-A assembly, we prepared tetR-EYFP fusion proteins to known functional domains of CENP-C (Carroll et al., 2010; Screpanti et al., 2011; Suzuki et al., 2004; Trazzi et al., 2009) (Fig. 7A). Tethering of domain II (amino acids 72–425), III (amino acids 426–537) or a fusion of domains VI and VII (VI+VII) (amino acids 760–943) were each found to be sufficient for *de novo* CENP-A assembly on the ectopic alphoid^tetO^ array (Fig. 7B,C).

We next checked whether these domains recruit CENP-I or M18BP1 (Fig. 7D–F). Tethering of domain II or VI+VII both led to recruitment of Halo–CENP-I or Halo–M18BP1 in 100% of cells. CENP-I interaction with CENP-C domain II is consistent with a previous *in vitro* analysis (Klare et al., 2015) and M18BP1 interaction with the C-terminus of CENP-C is consistent with a previous report (Dambacher et al., 2012). Tethering of domain II also recruited Halo–M18BP1 in 75% of cells, probably through CENP-I. We also observed interaction between CENP-C domain I (amino acids 1–71) and NSL1 (data not shown), which is consistent with previous *in vitro* analyses (Screpanti et al., 2011).

To test the involvement of CENP-I and M18BP1 in *de novo* CENP-A assembly by the various CENP-C domains, we repeated the above experiments after siRNA depletion of the two proteins (Fig. 7G). CENP-I depletion strongly decreased *de novo* CENP-A assembly on the ectopic alphoid^tetO^ array by the CENP-C domain II, but had no effect following tethering of full-length CENP-C or domain VI+VII. M18BP1 depletion dramatically reduced *de novo* CENP-A assembly following tethering of all of these fusion constructs. Unexpectedly, *de novo* CENP-A assembly by domain III depended on weak Halo–M18BP1 recruitment (Fig. 7F,G). CENP-A nucleosome binding by this domain has recently been reported to stabilize CENP-A nucleosomes (Falk et al., 2015; Kato et al., 2013). We confirmed that this CENP-A nucleosome-binding activity is also required for *de novo* CENP-A assembly on the ectopic site by point mutation of R522 (Fig. 7H). Thus the CENP-A recruitment caused by CENP-C domain III might not depend only on the interaction with M18BP1. Taken together, these results suggest that CENP-C induces *de novo* CENP-A assembly through at least two M18BP1 recruitment pathways: CENP-I recruitment by domain II and directly through its C terminus (Fig. 7I).

### Centromere assembly of newly synthesized CENP-A requires CENP-C and is enhanced via CENP-I

**Fig. 8.**
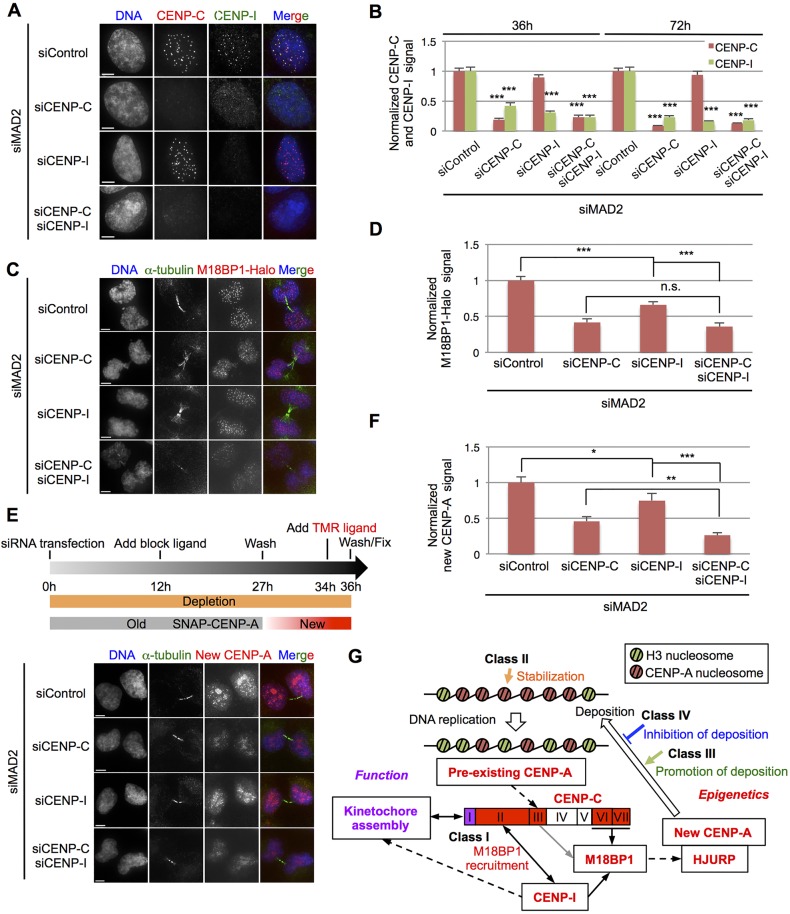
**Centromere assembly of newly synthesized CENP-A requires CENP-C and is enhanced through CENP-I.** (A) Representative images of HeLa-Int-03 cells transfected with control siRNA (siControl) or siRNA against CENP-C (siCENP-C), CENP-I (siCENP-i) or both, together with siRNA against MAD2 (siMAD2). Cells were stained with DAPI, anti-CENP-C (red) and anti-CENP-I (green) at 72 h after transfection. Scale bars: 5 μm. (B) Normalized CENP-C and CENP-I dot signals in each sample at 36 h and 72 h after siRNA transfection. ***P<0.001 (Mann–Whitney test). Results are mean±s.e.m. (*n*=39–48 cells). (C,E) Representative images of HeLa-Int-03 M18BP1–Halo cells (C) or HeLa-Int-03 SNAP–CENP-A cells (E) transfected with the indicated siRNAs. Cells were stained with DAPI, anti-α-tubulin (green) and the Halo-tag TMR Ligand (red) at 36 h after co-transfection (C). A schematic for the experiment in E is shown above the images. Pre-existing SNAP–CENP-A was quenched with block ligand until 9 h before fixation, and then newly synthesized SNAP–CENP-A was labeled with SNAP TMR Ligand before fixation at 36 h after siRNA transfection (E). Scale bars: 5 μm. (D,F) Normalized M18BP1–Halo (D) or normalized new CENP-A (F) dot signals in each sample. Asterisks indicate significant differences. *P<0.05; **P<0.01; ***P<0.001; n.s., not significant (Mann–Whitney test). Results are mean±s.e.m. (*n*=43–50 cells). (G) CENP-C and CENP-I coordinate centromere ‘function’ and ‘epigenetics’. Solid lines show recruitment activity observed in our present experiments and dashed lines show recruitment activity indicated previously. The CENP-C N-terminus assembles kinetochore through the Mis12 complex (function) and its C-terminus assembles newly synthesized CENP-A through M18BP1 (epigenetics). CENP-I recruited by the CENP-C domain II (amino acids 72–425) supports both pathways. Each class is also involved in maintenance of CENP-A assembly (see Discussion).

In order to check whether the conclusions of tethering experiments could be extended to CENP-A assembly at endogenous centromeres, we first tested the interdependency of kinetochore assembly of CENP-C and CENP-I. Interestingly, CENP-I levels at kinetochores fell dramatically after CENP-C depletion. In contrast, CENP-C levels at kinetochores were unaffected after 72 h of CENP-I depletion (Fig. 8A,B).

To ask whether CENP-C and CENP-I are required for the association of M18BP1 with centromeres, we depleted the two proteins in HeLa-Int-03 M18BP1-Halo cells, in which the endogenous M18BP1-encoding gene was fused with a Halo tag using CRISPR- and Cas-mediated genome editing. M18BP1 assembles on centromeres from telophase to early G1 (Fujita et al., 2007). We identified cells in early G1 phase by looking for small cells linked by α-tubulin in a mid-body. Depletion of either CENP-C or CENP-I caused a significant reduction in M18BP1–Halo signals at centromeres (to 42% or 66%, respectively) compared to the control (Fig. 8C,D).

Finally, we examined the assembly of newly synthesized CENP-A at centromeres during early G1 phase after depletion of CENP-C or CENP-I using HeLa-Int-03 SNAP-CENP-A cells stably expressing SNAP-tagged CENP-A. Newly synthesized SNAP–CENP-A was detected using quench-chase-pulse labeling (Jansen et al., 2007). Following CENP-C or CENP-I depletion, newly synthesized SNAP–CENP-A signals at centromeres were significantly reduced (to 44% or 75%, respectively) compared to the control (Fig. 8E,F). As the assembly of CENP-I at centromeres depends on CENP-C, the role of CENP-I for the newly synthesized CENP-A assembly is to enhance (reinforce) M18BP1 recruitment in parallel through interactions with CENP-C.

## DISCUSSION

Here, we have used synthetic biology (tethering) analysis to examine the role of a number of kinetochore proteins and chromatin-modifying enzymes in CENP-A assembly at a centromere and at an ectopic site on a chromosome arm. This analysis revealed four classes of factors that influence CENP-A assembly.

### Class I factors lead to M18BP1 recruitment through CENP-C and CENP-I

Class I factors are sufficient for *de novo* assembly of endogenous CENP-A on the ectopic alphoid^tetO^ array. CENP-C, CENP-I and components of the KMN network, a microtubule-binding complex, belong to this class (Fig. 4). In contrast, many other CENPs, which localize at centromeres across the cell cycle, did not induce substantial CENP-A assembly on the heterochromatic ectopic site, although CENP-L and CENP-O displayed a weak ability to mediate CENP-A assembly (Fig. 5B).

Tethering of KMN components induced *de novo* CENP-C assembly at the ectopic site. A similar result has been reported using tethering analysis that targeted these factors to the centrosome in *Drosophila* cells (Venkei et al., 2012). This process did not require CENP-A. Meanwhile, the number of CENP-A nucleosomes was reduced by half through DNA replication and until mitosis is completed. Our result suggests that interactions between CENP-C and the KMN network might reinforce reassembly or retention of CENP-C at the same site (Fig. 8G).

We found that CENP-C and CENP-I are sufficient for *de novo* CENP-A assembly upon the tethering of class I factors. Three CENP-C domains, from amino acids 72–425, 426–537 and 760–943, were sufficient for *de novo* CENP-A assembly (Fig. 7). The amino acid domain 760–943 seems to directly interact with M18BP1 as shown in *Xenopus* CENP-C (Moree et al., 2011). We also found that the domain comprising amino acids 72–425 can recruit M18BP1 through CENP-I (Figs 6 and 7). Therefore, it is quite reasonable that *de novo* CENP-A assembly by the tethering of class I factors is eventually dependent on M18BP1, which has been reported to be involved in recruitment of the CENP-A chaperon HJURP as part of the Mis18 complex (Barnhart et al., 2011; Wang et al., 2014).

### Class II factors support activities promoting CENP-A assembly

Class II factors increase CENP-A assembly on the HAC, but do not induce substantial *de novo* CENP-A assembly on the ectopic alphoid^tetO^ array. These are thought to be factors that cooperate with other activities present at the established HAC centromere to promote CENP-A assembly. Class II might include factors that do not directly recruit, but enhance the stability, assembly or activity of other factors that promote CENP-A assembly, including the Mis18 complex or HJURP.

MgcRacGAP and CENP-B reportedly stabilize CENP-A nucleosomes (Fachinetti et al., 2015; Fujita et al., 2015; Lagana et al., 2010). Thus, tethering these factors might increase CENP-A assembly by stabilizing CENP-A nucleosomes created by the canonical centromere assembly activity on the HAC but not at the ectopic site (Fig. 8G).

We have previously reported that CENP-B promotes heterochromatin formation on alphoid DNA integrated into chromosomal arms. However, CENP-B activity depends on chromatin context and it promotes *de novo* CENP-A assembly on the naked alphoid DNA introduced into cells (Okada et al., 2007). This suggests that CENP-B might also promote *de novo* CENP-A assembly in addition to stabilizing CENP-A nucleosomes at the HAC centromere.

### Class III factors may form chromatin states promoting histone exchange

Class III factors induce robust *de novo* CENP-A assembly on the ectopic alphoid^tetO^ array in cells overexpressing CENP-A. All tested HATs belong to this class. Transient acetylation of H3 occurs at centromeres during early G1 phase and inhibition of this activity by Suv39h1 tethering leads to decreased CENP-A assembly (Ohzeki et al., 2012). The class III factors SSRP1 and RSF1 are general histone chaperones involved in transcription (LeRoy, 1998) but have also been shown to localize at centromeres (Chen et al., 2015; Okada et al., 2009; Perpelescu et al., 2009). SSRP1 is a component of the ‘facilitates chromatin transcription’ (FACT) complex, which promotes histone exchange by evicting histones ahead of polymerases and redepositing them after transcription or replication has occurred (Belotserkovskaya et al., 2003; Formosa, 2013).

When Halo-tagged canonical histone H3 instead of Halo–CENP-A was expressed, class III factors also induced Halo–H3 assembly at the ectopic site. Assembly of H3.3 and CENP-A were more efficiently induced on the alphoid^tetO^ array than H3.1 following the tethering of class III factors. H3.3 is normally deposited by histone exchange coupled with chromatin remodeling and transcription independently of DNA replication (Gurard-Levin et al., 2014; Tagami et al., 2004; Wirbelauer et al., 2005). Thus, CENP-A deposition might exploit similar mechanisms. Indeed, H3.3 has been suggested to be a placeholder for CENP-A until its replenishment in early G1 human cells (Dunleavy et al., 2011). We suggest that class III factors induce CENP-A or H3.3 assembly (depending on the expressed histones) by changing the chromatin state to promote histone exchange, possibly by locally inducing transcription (Fig. 8G).

Other class III factors, including the Mis18 complex, CENP-H and SKA1 are centromere- or kinetochore-specific factors and not known to have direct functions in establishing chromatin states. These factors might promote CENP-A assembly by recruiting other general chromatin factors. Thus, they are potential candidates to investigate the mechanism by which centromere-specific and general chromatin factors coordinately regulate centromere chromatin.

### Class IV factors might inhibit CENP-A assembly through repression of transcription

Class IV factors decrease CENP-A levels on the HAC and do not induce robust *de novo* CENP-A assembly on the ectopic site. Many of the HMTs and HDACs involved in transcriptional silencing belong to this class. Thus, we propose that class IV factors inhibit CENP-A deposition on the HAC through modulating heterochromatin formation and repression of histone exchange, in contrast to class III factors, which induce chromatin opening and exchange (Fig. 8G).

HDACs were found to decrease CENP-A signals on the HAC. This is consistent with our previous observation that treatment with the HDAC inhibitor trichostatin A induced CENP-A assembly on a heterochromatinized ectopic site (Nakano et al., 2003; Okamoto et al., 2007). The results of the present study strongly support our notion that centromere formation and heterochromatin formation (e.g. transcriptional silencing) are in balance and antagonize each other (Bergmann et al., 2012; Ohzeki et al., 2012, 2015; Okada et al., 2007).

### Interactions of class I, II and III factors in CENP-A assembly at centromeres

Following from the discussion above, we suggest that, other than recruitment of CENP-A, basic histone exchange reactions to deposit CENP-A by class III factors and subsequent stabilization of deposited CENP-A nucleosome by class II factors are also required for CENP-A assembly (Fig. 8G). Indeed, many factors of these classes have been reported to be required for CENP-A assembly at endogenous centromeres. CENP-H (class III) and CENP-I (class I) form a complex at the centromere and are reported to be required for newly synthesized CENP-A assembly (Okada et al., 2006). Thus, class I factors, including CENP-C and CENP-I, might recruit not only CENP-A through M18BP1, but also these activities to change chromatin states (class III) on the ectopic site and to stabilize *de novo* assembled CENP-A nucleosomes (class II).

### M18BP1 activity is dependent upon CENP-C and CENP-I

M18BP1 is required for *de novo* CENP-A assembly induced by CENP-C and/or CENP-I. It might therefore be expected that tethering of M18BP1 would bypass the requirement for CENP-C or CENP-I in *de novo* CENP-A assembly. Surprisingly, tethering of tetR-EYFP–M18BP1 on its own did not induce *de novo* assembly of endogenous CENP-A on the ectopic alphoid^tetO^ array. This suggests that CENP-C, CENP-I or some interacting factor(s) might be involved in regulating or reinforcing M18BP1 activity. Indeed, M18BP1 tethering increased endogenous CENP-A assembly on the HAC centromere where CENP-C and CENP-I are pre-assembled (Fig. 4). Subsequent studies will reveal whether the downstream factors are involved in phosphorylation (McKinley and Cheeseman, 2014; Silva et al., 2012) or other modifications of M18BP1.

### The hierarchy of CENP-C and CENP-I is subtly different between species

In chicken cells (DT40), the centromere localization of CENP-C is reported to depend on CENP-I (Nishihashi et al., 2002) and newly synthesized CENP-A assembly on centromeres depends on CENP-I rather than CENP-C (Okada et al., 2006). By contrast, in human cells (HeLa), we found that the centromere assembly of CENP-I depends on CENP-C and that CENP-I has a role in enhancing newly synthesized CENP-A assembly through M18BP1 assembly downstream of CENP-C. This suggests that the hierarchy of these two factors is subtly different between these species, although both CENP-C and CENP-I are conserved factors for centromere epigenetics.

### CENP-C and CENP-I coordinate centromere ‘function’ and ‘epigenetics’

CENP-C is widely conserved from yeasts to higher eukaryotes (Przewloka et al., 2011; Talbert et al., 2004), but is not present in some insects carrying holocentromeres lacking CENP-A. This suggests that a primary conserved function of CENP-C is recognition of CENP-A nucleosomes (Drinnenberg et al., 2014). In this study, we found that CENP-C can induce kinetochore formation coupled with *de novo* CENP-A chromatin assembly. Thus, CENP-C binding to CENP-A nucleosomes recruits both kinetochore components and also CENP-A molecules. CENP-I is also important for kinetochore assembly (Basilico et al., 2014; Cheeseman et al., 2008; Hori et al., 2003; Liu et al., 2003; Nishihashi et al., 2002), and indeed we observed mitotic arrest upon CENP-I depletion (Fig. S4A). CENP-C, in parallel with CENP-I enhancement (reinforcement), thereby coordinates two important mechanisms required for accurate chromosome segregation, kinetochore and epigenetic centromere maintenance or memory (Fig. 8G).

## MATERIALS AND METHODS

### Cell lines

HeLa-HAC-2-4 is a derivative of HeLa-HAC-R5. The HeLa-HAC-R5 and HeLa-Int-03 cell line were previously described (Ohzeki et al., 2012). HeLa-HAC-2-4 ETY3-Alone, tetR-EYFP–HJURP or tetR-EYFP–Suv39h1 cell lines, which stably expresses tetR-EYFP alone, or tetR-EYFP fused with HJURP or Suv39h1, respectively, were established uisng the Jump-in integration system (Life Technologies) for transfection of each pJETY3 plasmid into HeLa-HAC-2-4 cells. HeLa-Int-03 SNAP-CENP-A cells, which stably express SNAP–CENP-A, were established by the Jump-in integration of a pJETY3-based SNAP–CENP-A-expressing plasmid into HeLa-Int-03 cells. HeLa-Int-03 M18BP1-Halo cells, which stably express M18BP1–Halo, were established by tagging with Halo tag at the C terminus of the *M18BP1* locus using a CRISPR- and Cas-mediated genome engineering. Paired single-guide RNA-expressing vectors targeting 5′-AAATGAGAAAATATGATTCC-3′ and 5′-TGCTTTATGGTAAAAATCCC-3′ in the *M18BP1* locus were prepared. The 5′ or 3′ homology arm for the *M18BP1* locus was amplified using the following primer sets 5′-CTCTAGAGGATCCCCGAGCATGTACTTCAAACTTGCC-3′ and 5′-TGCAGAATCAGAGTTCGAAAAATAATAATC-3′, or 5′-GTTTGTATTTTCAACTGGAGTACATG-3′ and 5′-GCCAGTGAATTCGAGTATGTCAAGTTTCAAAACATAACAAGC-3′. Single-guide RNA-expressing vectors, donor vector including the Halo tag and puromycin-resistant gene with homology arms, and Cas9 nickase mutant (D10A)-expressing vector were co-transfected into HeLa-Int-03 cells and selected with 0.4 μg/ml puromycin.

### Cell culture and transfection

HeLa cell lines were grown in Dulbecco's modified Eagle's medium (Nacalai Tesque) supplemented with 10% fetal bovine serum (FBS) at 37°C in a 5% CO_2_ atmosphere. For transfections, Lipofectamin 2000 (Invitrogen) or FuGENE HD (Promega) was used for siRNA or usual plasmid vectors, respectively. siRNAs were transfected twice at a 10-h interval for efficient depletion. The following plasmid transfection was performed after 24 h from the first siRNA transfection. siRNA sequence for M18BP1 was described in Fujita et al. (2007) and synthetized by Ambion. siRNAs for CENP-A (s2908), CENP-C (s2912), CENP-I (s5375), MAD2 (s8393) and Control (AM4635) were obtained from Ambion.

### Cell staining

Indirect immunofluorescent staining was performed as previously described (Ohzeki et al., 2012) with slight modifications. Antibodies used in this study are shown in Table S1. For Halo tag staining, cells expressing Halo-tag fusion proteins were treated with 2 nM Halo-tag TMR Ligand (Promega) for 12 h before fixation. For SNAP–CENP-A staining, cells were treated with 1 μM SNAP-cell-Block (NEB) to block the SNAP tag from 24 to 9 h before fixation and washed. 0.6 μM SNAP-Cell TMR-Star (NEB) was added 2 h before fixation and washed out 30 min before fixation. For chromosome spreads and fluorescence in situ hybridization (FISH), cells were treated with 350 nM of TN-16 (WAKO) (Kitagawa et al., 1995) for 6 h in the growth medium. Mitotic cells were harvested by pipetting, incubated in a hypotonic buffer (20 mM Tris-HCl pH 7.4, 1 mM EGTA and 40 mM KCl) for 10 min on ice and then spread on cover glass by Cytospin3 (Shandon). The subsequent immunostaining and FISH were carried out according to a previously described method (Ikeno et al., 1998; Ohzeki et al., 2002).

### Plasmids expressing Halo and tetR-EYFP fusion proteins

For Halo fusion protein expression, Flexi Halo tag clones (Kazusa DNA research institute, http://www.kazusa.or.jp/kop/dsearch-e/) were used. Each gene is cloned into the C-terminus of the Halo tag. pJETY3, pJETY3-HJURP, -Suv39h1 or -Mis18α, which express each tetR-EYFP fusion protein, were as previously described in Ohzeki et al. (2012). For other tetR-EYFP fusions, PCR product or cDNA fragment cut out from each Flexi Halo tag clone vector was cloned into pJETY3.

### Microscopy

For quantification analysis, *z*-stack images covering an entire nuclear signal were acquired on an Axio Observer.Z1 (Zeiss) microscope equipped with a CSU-X1 confocal scanner unit (Yokogawa), iXon3 DU897E-CS0 camera (Andor) and Plan-Apochromat 100×/1.46 oil lens (Zeiss) with a spacing of 0.22 μm using Andor iQ2 software (Andor). Images in Fig. 1C,E and Fig. 8A,C,E were acquired by same method. Other images were acquired on an Axio Observer.Z1 (Zeiss) equipped with a LSM700 scanning module and an Objective Plan-Apochromat 63×/1.46 oil lens (Zeiss) using ZEN 2009 software (Zeiss). *z*-stack images were acquired with a spacing of 0.38 μm. Images shown are maximum projection of several slices around the ectopic alphoid^tetO^ integration site or whole nucleus. Counting assays were performed in the same microscopic condition. Visible signals on the ectopic site in interphase cells were counted.

### Quantification of images

ImageJ (National Institutes of Health) was used for quantification. Before quantification, unequal illumination derived from optical system was corrected using the image of a uniform fluorescent. For HAC analysis (Fig. 1), the sum of the intensity of CENP-A signal on the alphoid^tetO^-HAC and endogenous centromeres in the same nucleus was measured (see Fig. S1A,B). The CENP-A signal on the HAC was normalized to the average CENP-A signal on endogenous centromeres. For endogenous centromeres analysis (Fig. 8), the sum of the intensity and the number of dot signals in the same nucleus were measured and calculated as the average of dot signals.

### Immunoblotting and cell fractionation

Whole-cell extracts were prepared by lysing cells in Laemmli sample buffer (Bio-Rad) after 5 min incubation with 50 U/ml Pierce Universal Nuclease for Cell Lysis (Thermo) and 0.1% NP40 in PBS. Samples were separated by SDS-PAGE using Mini-PROTEAN TGX precast gels (Bio-Rad) and transferred onto membranes using Trans-Blot Turbo Transfer Pack (Bio-Rad). The membrane was blocked with 3% BSA and 0.1% Tween 20 in PBS and incubated with primary antibodies (in 1% BSA and 0.1% Tween 20 in PBS) overnight at 4°C and with secondary antibodies (with 1% low-fat milk and 0.1% Tween 20 in PBS) for 1 h at room temperature. Antibodies used in this study are shown in Table S1. SuperSignal West Femto Maximum Sensitivity Substrate (Thermo) was used for detection. For cell fractionation, cells were homogenized by passing through a G27 needle syringe 20 times in ice-cold lysis buffer [3.75 mM Tris-HCl pH 7.5, 20 mM KCl, 0.5 mM EDTA, 0.5 mM DTT, 0.05 mM spermidine, 0.125 mM spermine, 0.1% NP40 and a 1/100 volume of Protease Inhibitor Cocktail (Sigma)]. A one-quarter volume of 1.5 M NaCl prepared in lysis buffer was added to the lysates and incubated for 30 min on ice. The lysates were centrifuged at 12,000 ***g*** for 10 min to fractionate supernatants and pellets. Laemmli sample buffer was added to supernatants. Pellets were washed once in PBS and lysed with Laemmli sample buffer and sonicated with a Bioruptor (Cosmo Bio) machine.
